# Prognostic significance of CHADS_2_ and CHA_2_DS_2_-VASc scores to predict unfavorable outcomes in hospitalized patients with COVID-19

**DOI:** 10.34172/jcvtr.2022.07

**Published:** 2022-03-14

**Authors:** Mahnaz Montazeri, Mohammad Keykhaei, Sina Rashedi, Shahrokh Karbalai Saleh, Marzieh Pazoki, Azar Hadadi, Seyyed Hamidreza Sharifnia, Mehran Sotoodehnia, Sanaz Ajloo, Samira Kafan, Haleh Ashraf

**Affiliations:** ^1^Department of Infectious Diseases, Sina Hospital, Tehran University of Medical Sciences, Tehran, Iran; ^2^Research Development Center, Sina Hospital, Tehran University of Medical Sciences, Tehran, Iran; ^3^Department of Cardiology, Sina Hospital, Tehran University of Medical Sciences, Tehran, Iran; ^4^Department of Pulmonary Medicine, Sina Hospital, Tehran University of Medical Sciences, Tehran, Iran; ^5^Research Center For Clinical Virology, Tehran University of Medical Sciences, Tehran, Iran; ^6^Department of Anesthesiology, Sina Hospital, Tehran University of Medical Sciences, Tehran, Iran; ^7^Department of Emergency Medicine, Sina Hospital, Tehran University of Medical Sciences, Tehran, Iran; ^8^Cardiac Primary Prevention Research Center (CPPRC), Cardiovascular Diseases Research Institute, Tehran University of Medical Sciences, Tehran, Iran

**Keywords:** Acute Kidney Injury, Acute Respiratory Distress Syndrome, Cardiac Injury, COVID-19, Mortality

## Abstract

***Introduction:*** Owing to the imposed burden of the coronavirus disease 2019 (COVID-19),the need for stratifying the prognosis of patients has never been timelier. Hence, we aimed to ascertain the value of CHADS_2_, CHA_2_DS_2_-VASc, and CHA_2_DS_2_-VASc-M (one point for male instead of female) scores to predict unfavorable outcomes in COVID-19 patients.

***Methods:*** We enrolled consecutive patients above 18 years of age with confirmed COVID-19,who were admitted between February 16 and November 1, 2020. The primary endpoint of this study was three-month all-cause mortality. The secondary endpoints were considered four major in-hospital clinical features, including acute respiratory distress syndrome, cardiac injury,acute kidney injury, and mechanical ventilation.

***Results:*** A total of 1,406 hospitalized COVID-19 patients were studied, among which 301(21.40%) patients died during the follow-up period. Regarding the risk scores, CHADS _2_≥1,CHA_2_DS_2_-VASc≥2, and CHA_2_DS_2_-VASc-M≥2 were significantly associated with mortality. The performance of all risk scores for predicting mortality was satisfactory (area under the curve:0.668, 0.668, and 0.681, respectively). Appraising secondary endpoints, we found that all three risk scores were associated with increased risk of acute respiratory distress syndrome, cardiac injury, acute kidney injury, and mechanical ventilation. Lastly, we revealed that all risk scores were significantly correlated with serum levels of laboratory biomarkers.

***Conclusion:*** Our analysis illustrated that the CHADS_2_, CHA2DS_2_-VASc, and CHA_2_DS_2_-VASc-Mscores could aid prognostication of unfavorable outcomes in COVID-19 patients. Therefore,these easily calculable methods could be integrated into the overall therapeutic strategy to guide the COVID-19 management more accurately.

## Introduction

As a tremendous challenge and threat to public health, the novel coronavirus disease 2019 (COVID-19) attracts increasing attention.^1^ By April 17, 2021, the COVID-19 had affected more than 141 million people, claiming more than 3.0 million lives.^2^ Although respiratory failure has remained the most common culprit for unfavorable outcomes, the COVID-19 has been evidenced to be a complex condition with multiorgan involvement.^3^ Appraising the determinants of the disease progression, cardiovascular (CV) risk factors have played a pivotal role in the clinical course of patients with COVID-19.^4^ In this perspective, practical prognostication and early identification of critically ill patients with COVID-19 may aid in optimizing the allocation of healthcare resources and delivering proper treatment.^1,5^ However, no single prognostic model has represented incremental value for timely risk stratification of the disease. Hence, it seems imperative to develop robust and straightforward methods to ascertain the prognosis of patients with COVID-19.

The CHADS_2_ and CHA_2_DS_2_-VASc scores are well-validated clinical prediction tools, commonly applied to identify the risk of thromboembolic events in patients with atrial fibrillation (AF).^6^ Besides, owing to the clusters of stroke and cardiovascular risk factors included within the CHADS_2_ and CHA_2_DS_2_-VASc scores, the privilege of using these methods for predicting thromboembolism and mortality beyond the original disease has been well identified.^6-8^ In support of this concept, Chen and colleagues^7^ indicated the essential impact of CHADS_2_ and CHA_2_DS_2_-VASc scores on predicting one-year all-cause mortality in patients with systolic heart failure. Across patients with COVID-19, each of the component comorbidities of the aforementioned scores has been independently associated with an increased risk of mortality.^9-14^ In addition, the simplicity of calculating these risk scores could facilitate their adoption in chaotic settings during the COVID-19 pandemic.^15^ Therefore, it can be intuitive to hypothesize that mortality may also be well captured by the CHADS_2_ and CHA_2_DS_2_-VASc scores in patients with COVID-19.

So far, few studies have narrowed the path, linking CHADS_2_ and CHA_2_DS_2_-VASc scores with poor outcomes in patients with COVID-19.^15-17^ However, it seems difficult to arrive at the best evidence-based decision concerning the current literature as different outcomes rather than mortality have not been adequately evaluated. Furthermore, longitudinal data regarding the impact of these risk scores on COVID-19 patients are still lacking. Thus, we sought to ascertain the value of CHADS_2_ and CHA_2_DS_2_-VASc scores, as well as their refinement form, the CHA_2_DS_2_-VASc-M score, to predict all-cause mortality in COVID-19 patients. Secondly, we added new insights to the existing literature by appraising whether these scores could be used to estimate the susceptibility to develop acute respiratory distress syndrome (ARDS), cardiac injury, acute kidney injury (AKI), and mechanical ventilation in patients with COVID-19.

## Materials and Methods

### Ethical considerations

The research was conducted according to the principles of the 1975 declaration of Helsinki. All patients gave written informed consent before inclusion in the study.

### Study design and participants

We enrolled consecutive patients above 18 years of age with laboratory or radiologically confirmed COVID-19 who were admitted to our tertiary center between February 16 and November 1, 2020. The inclusion criteria were as follows: 1. Patients with positive real-time reverse-transcriptase polymerase-chain-reaction (PCR) test of respiratory specimens for severe acute respiratory syndrome coronavirus 2 (SARS-CoV-2); or 2. Patients clinically suspicious for COVID-19 based on the World Health Organization (WHO) interim guidance with a chest computed tomography (CT) involvement in favor of COVID-19.^18^ The clinical and laboratory data of the recruited patients were attained from the electronic medical records and reviewed thoroughly to ensure accuracy. The discharged patients were followed for at least three months from the day of admission to assess the mortality.

### Definitions and endpoints

The CHADS_2_ score was ascertained for each patient accordingly: congestive heart failure (CHF) (1 point), hypertension (1 point), age ≥ 75 (1 point), diabetes mellitus (DM) (1 point), and previous stroke or transient ischemic attack (TIA) (2 points). The CHA_2_DS_2_-VASc score for every patient was calculated by assigning 1 point for CHF, hypertension, age 65 to 74 years, DM, vascular disease, female sex, and 2 points for age ≥ 75, and previous stroke/TIA. Given that the male sex has been recognized as a risk factor for the severity and poor prognosis of COVID-19,^19^ we also calculated a modified CHA_2_DS_2_-VASc score (CHA_2_DS_2_-VASc-M) by giving 1 point for the male sex instead of the female sex.

The primary endpoint of this study was three-month all-cause mortality. The secondary endpoints were considered as four major in-hospital clinical features: 1. ARDS determined in adherence with Berlin definition;^20^ 2. Cardiac injury established as the elevated serum level of highly sensitive cardiac troponin I (hs-cTnI) above the 99th centile upper reference limit (26 pg/mL for males and 11 pg/mL for females);^21^ 3. AKI defined according to the KDIGO criteria;^22^ 4. Mechanical ventilation with endotracheal intubation performed in patients with progressive hypoxemic respiratory failure failing to respond to standard non-invasive oxygen therapy.^18^

### Statistical analysis

Normally distributed continuous variables were presented as mean ± standard deviation and compared using the independent samples T-test. Categorical variables were summarized as counts and percentages and compared utilizing the chi-squared test. Kaplan-Meier survival curves were plotted to investigate the prognostic significance of CHADS_2_, CHA_2_DS_2_-VASc, and CHA_2_DS_2_-VASc-M scores regarding the three-month mortality (the primary endpoint). Furthermore, Cox proportional hazard regression was performed for each of these risk scores and their components (CHF, hypertension, age 65-74 years, age ≥ 75, DM, stroke or TIA, vascular disease, and female gender) in univariate analysis, and hazard ratios (HRs) and their corresponding 95% confidence intervals (CIs) were calculated. The category with a score of zero for each risk score was set as the reference category. Additionally, the multivariate analyses for the risk scores were conducted adjusting for other comorbidities, including dyslipidemia, atrial fibrillation, chronic kidney disease, chronic respiratory disease, malignancy, tobacco smoking, and opium consumption.

The univariate and multivariate logistic regression analyses (adjusting for the mentioned comorbidities) were performed concerning the secondary endpoints, and the odds ratios (ORs) and 95% CIs were calculated. Because of the small number of patients with high scores, patients with CHADS_2_ ≥ 3, CHA_2_DS_2_-VASc ≥ 4, and CHA_2_DS_2_-VASc-M ≥ 4 were combined. The prediction performance of these three risk scores for the primary and secondary endpoints was investigated by receiver operating characteristic (ROC) curves and calculation of the area under the curve (AUC) with the corresponding 95% CIs, and compared using the “roccomp” command. Ultimately, Spearman rank correlation was utilized to measure the degree of association between the risk scores and serum levels of three laboratory parameters: 1. C-reactive protein (CRP) (mg/L), 2. Hs-cTnI (pg/mL), and 3. D-dimer (ng/mL). All statistical analyses were conducted utilizing Stata (version 14.2; Stata Corp, College Station, Texas, USA), with *P* values less than 0.05 indicating statistical significance.

## Results

### Patient characteristics

After excluding seven patients below 18 years of age, a total of 1,406 hospitalized COVID-19 patients were included in this study. The diagnosis of COVID-19 was determined based on PCR test and chest CT in 832 (59.17%) and 574 (40.83%), respectively. Table 1 summarizes the demographics, comorbidities, medications, CHADS_2_, CHA_2_DS_2_-VASc, CHA_2_DS_2_-VASc-M scores, and in-hospital outcomes of the study cohort. The participants’ mean age was 59.47 ± 16.48, and males accounted for 60.46% (850/1,406) of the patients.

**Table 1:** Characteristics of the hospitalized COVID-19 patients based on three-month mortality

	**Total patients (n=1,406)**	**Non-survivors (n=301; 21.40%)**	**Survivors (n=1,105; 78.60%)**	***P*** ** value**
Demographics				
Age (years)	59.47 ± 16.48	69.34 ± 13.99	56.78 ± 16.08	< 0.001^*^
Male gender	850 (60.46%)	188 (62.46%)	662 (59.91%)	0.423
BMI (kg/m^2^)	27.46 ± 4.75	27.04 ± 4.81	27.54 ± 4.73	0.249
Comorbidities				
Hypertension	638 (45.38%)	177 (58.80%)	461 (41.72%)	< 0.001^*^
DM	423 (30.09%)	111 (36.88%)	312 (28.24%)	0.004^*^
Dyslipidemia	497 (35.35%)	139 (46.18%)	358 (32.40%)	< 0.001^*^
Coronary artery disease	300 (21.34%)	83 (27.57%)	217 (19.64%)	0.003^*^
CHF	79 (5.62%)	40 (13.29%)	39 (3.53%)	< 0.001^*^
Atrial fibrillation	31 (2.20%)	14 (4.65%)	17 (1.54%)	0.001^*^
Stroke/TIA	64 (4.55%)	28 (9.30%)	36 (3.26%)	< 0.001^*^
CKD	69 (4.91%)	23 (7.64%)	46 (4.16%)	0.013^*^
Chronic respiratory disease	91 (6.47%)	26 (8.64%)	65 (5.88%)	0.085
Malignancy	64 (4.55%)	29 (9.63%)	35 (3.17%)	< 0.001^*^
Tobacco smoking	169 (12.02%)	42 (13.95%)	127 (11.49%)	0.245
Opium consumption	92 (6.54%)	24 (7.97%)	68 (6.15%)	0.285
CHADS_2_				
0	549 (39.05%)	59 (19.60%)	490 (44.34%)	< 0.001^*^
1	414 (29.45%)	91 (30.23%)	323 (29.23%)	
2	280 (19.91%)	79 (26.25%)	201 (18.19%)	
≥ 3	163 (11.59%)	72 (23.92%)	91 (8.24%)	
CHA_2_DS_2_-VASc				
0	290 (20.63%)	31 (10.30%)	259 (23.44%)	< 0.001^*^
1	352 (25.04%)	49 (16.28%)	303 (27.42%)	
2	245 (17.43%)	49 (16.28%)	196 (17.74%)	
3	197 (14.01%)	48 (15.95%)	149 (13.48%)	
≥ 4	322 (22.90%)	124 (41.20%)	198 (17.92%)	
CHA_2_DS_2_-VASc-M				
0	175 (12.45%)	14 (4.65%)	161 (14.57%)	< 0.001^*^
1	402 (28.59%)	46 (15.28%)	356 (32.22%)	
2	259 (18.42%)	54 (17.94%)	205 (18.55%)	
3	219 (15.58%)	60 (19.93%)	159 (14.39%)	
≥ 4	351 (24.96%)	127 (42.19%)	224 (20.27%)	
In-hospital medications				
Hydroxychloroquine	719 (51.14%)	120 (39.87%)	599 (54.21%)	< 0.001^*^
Lopinavir/ritonavir	534 (37.98%)	122 (40.53%)	412 (37.29%)	0.304
Favipiravir	160 (11.38%)	37 (12.29%)	123 (11.13%)	0.574
Atazanavir	180 (12.80%)	44 (14.62%)	136 (12.31%)	0.288
Remdesivir	120 (8.53%)	43 (14.29%)	77 (6.97%)	< 0.001^*^
Umifenovir	102 (7.25%)	19 (6.31%)	83 (7.51%)	0.477
Interferon β-1a	384 (27.31%)	97 (32.23%)	287 (25.97%)	0.031^*^
Azithromycin	123 (8.75%)	21 (6.98%)	102 (9.23%)	0.220
Steroids	663 (47.16%)	179 (59.47%)	484 (43.80%)	< 0.001^*^
In-hospital clinical features				
ARDS	383 (27.24%)	190 (63.12%)	193 (17.47%)	< 0.001^*^
Cardiac injury	317 (22.55%)	148 (49.17%)	169 (15.29%)	< 0.001^*^
AKI	177 (12.59%)	121 (40.20%)	56 (5.07%)	< 0.001^*^
Mechanical ventilation	168 (11.95%)	157 (52.16%)	11 (1.00%)	< 0.001^*^

Abbreviations: AKI, acute kidney injury; ARDS, acute respiratory distress syndrome; BMI, body mass index; CHF, congestive heart failure; CKD, chronic kidney disease; DM, diabetes mellitus; RAAS, renin-angiotensin-aldosterone system; TIA, transient ischemic attack Continuous variables are presented as mean ± standard deviation, categorical variables as number (%). *P < 0.05 is significant.

### Three-month mortality

**Figure 1:**
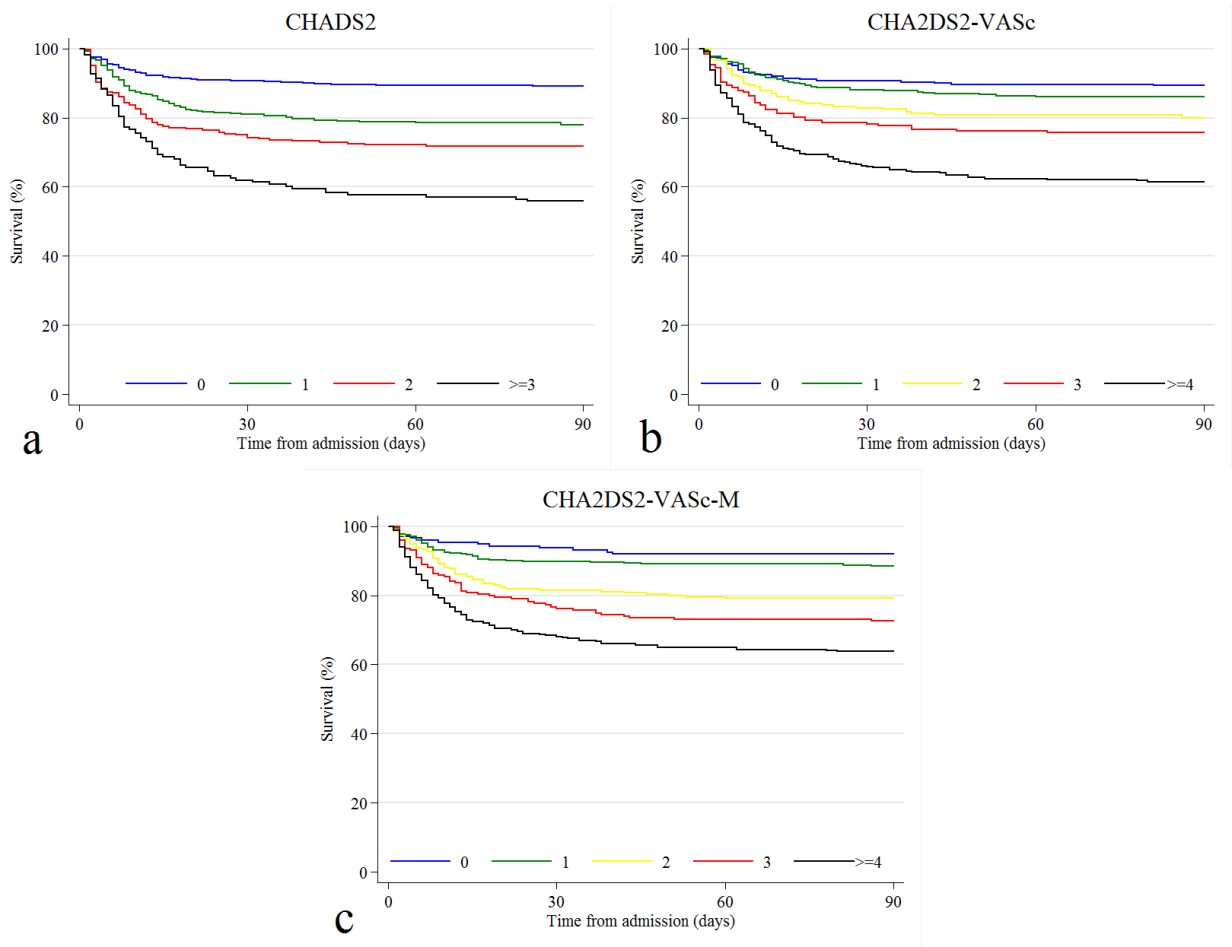
Kaplan-Meier survival curves for three-month mortality regarding (a) CHADS_2_, (b) CHA_2_DS_2_-VASc, and (c) CHA_2_DS_2_-VASc-M scores

**Figure 2:**
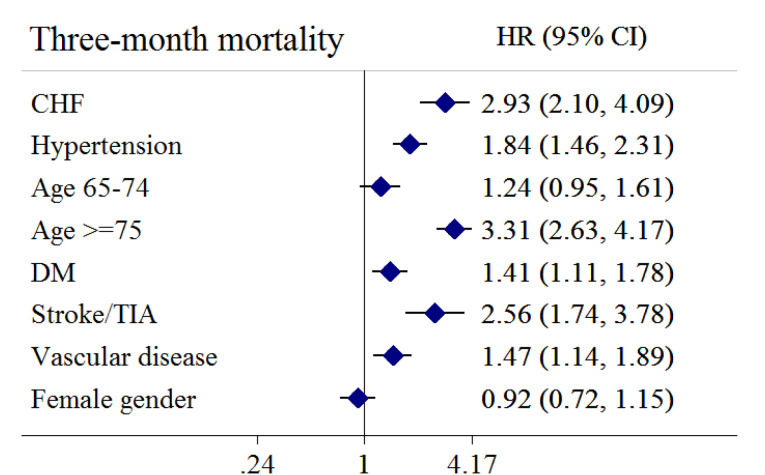
Forrest plot of hazard ratios for three-month mortality of individual CHA_2_DS_2_-VASc components

**Figure 3:**
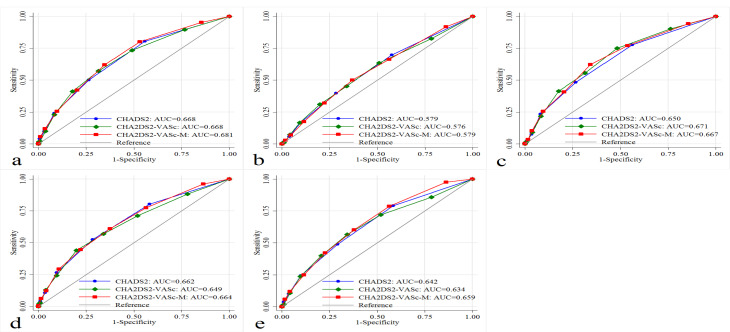
Predictive performance of CHADS_2_, CHA_2_DS_2_-VASc, and CHA_2_DS_2_-VASc-M scores regarding (a) Three-month mortality, (b) ARDS, (c) Cardiac injury, (d) AKI, and (e) Mechanical ventilation. Abbreviations: AKI, acute kidney injury; ARDS, acute respiratory distress syndrome.

During the follow-up period, 301 (21.40%) patients died. Compared to the survivors, the deceased patients were older (*P* < 0.001) and had a higher percentage of hypertension (*P* < 0.002), DM (*P* = 0.004), dyslipidemia (*P* < 0.001), coronary artery disease (*P* = 0.003), CHF (*P* < 0.001), atrial fibrillation (*P* = 0.001), stroke/TIA (*P* < 0.001), chronic kidney disease (CKD) (*P* = 0.013), and malignancy (*P* < 0.001). Non-survivors were more likely to receive remdesivir, interferon-β1a, and steroids and less hydroxychloroquine (Table 1). Regarding the risk scores, CHADS_2_ ≥ 1, CHA_2_DS_2_-VASc ≥ 2, and CHA_2_DS_2_-VASc-M ≥ 2 were associated with mortality (Table 2) (Figure 1). The mortality rates for patients with CHADS_2_ score of 0, 1, 2, and ≥ 3 were 10.74%, 21.98%, 28.21%, 44.17%, respectively. Similar trends of mortality rates were observed regarding CHA_2_DS_2_-VASc and CHA_2_DS_2_-VASc-M risk scores. Except for age 65-74 years and female gender, all the components of these scores were correlated with mortality (Figure 2). Based on ROC curves, CHADS_2_, CHA_2_DS_2_-VASc, and CHA_2_DS_2_-VASc-M reached the AUC of 0.668 (95% CI 0.635 - 0.701), 0.668 (95% CI 0.634 - 0.702), 0.681 (95% CI 0.648 - 0.714), respectively (Figure 3a). No statistically significant difference was observed between these three AUCs (*P* = 0.250) (Supplementary Table S1).

**Table 2:** Results of the univariate and multivariate analyses for three-month mortality and in-hospital outcomes of the hospitalized COVID-19 patients regarding the three risk scores

	**Univariate analysis**			Multivariate analysis*		
	**ES**	**95% CI**	***P*** **-value**	**ES**	**95% CI**	***P*** ** value**
Three-month mortality						
CHADS_2_						
0	Reference			Reference		
1	2.154	1.552 – 2.989	< 0.001	2.182	1.564 – 3.043	< 0.001^*^
2	2.923	2.086 – 4.096	< 0.001	2.754	1.915 – 3.960	< 0.001^*^
≥ 3	4.929	3.492 – 6.956	< 0.001	4.626	3.151 – 6.791	< 0.001^*^
CHA_2_DS_2_-VASc						
0	Reference			Reference		
1	1.308	0.834 – 2.051	0.242	1.281	0.814 – 2.015	0.284
2	1.943	1.239 – 3.047	0.004	1.994	1.261 – 3.155	0.003^*^
3	2.469	1.572 – 3.879	< 0.001	2.453	1.537 – 3.917	< 0.001^*^
≥ 4	4.226	2.850 – 6.265	< 0.001	4.059	2.623 – 6.280	< 0.001^*^
CHA_2_DS_2_-VASc-M						
0	Reference			Reference		
1	1.459	0.802 – 2.655	0.215	1.156	0.831 – 2.765	0.175
2	2.779	1.543 – 5.002	0.001	2.735	1.512 – 4.947	0.001^*^
3	3.781	2.113 – 6.765	< 0.001	3.692	2.039 – 6.688	< 0.001^*^
≥ 4	5.342	3.076 – 9.279	< 0.001	5.050	2.849 – 8.949	< 0.001^*^
ARDS						
CHADS_2_						
0	Reference			Reference		
1	1.435	1.066 – 1.932	0.017	1.418	1.046 – 1.922	0.024^*^
2	1.710	1.236 – 2.367	0.001	1.651	1.155 – 2.360	0.006^*^
≥ 3	2.413	1.685 – 3.511	< 0.001	2.274	1.495 – 3.457	< 0.001^*^
CHA_2_DS_2_-VASc						
0	Reference			Reference		
1	0.870	0.598 – 1.267	0.470	0.852	0.583 – 1.244	0.408
2	1.331	0.902 – 1.964	0.149	1.275	0.855 – 1.902	0.233
3	1.256	0.829 – 1.904	0.281	1.201	0.778 – 1.855	0.407
≥ 4	1.951	1.368 – 2.781	< 0.001	1.853	1.233 – 2.783	0.003^*^
CHA_2_DS_2_-VASc-M						
0	Reference			Reference		
1	1.497	0.954 – 2.348	0.079	1.551	0.985 – 2.440	0.058
2	1.461	0.903 – 2.366	0.122	1.487	0.912 – 2.423	0.111
3	2.136	1.320 – 3.458	0.002	2.093	1.269 – 3.451	0.004^*^
≥ 4	2.505	1.604 – 3.913	< 0.001	2.426	1.506 – 3.907	< 0.001^*^
Cardiac injury						
CHADS_2_						
0	Reference			Reference		
1	1.950	1.388 – 2.739	< 0.001	1.571	1.101 – 2.241	0.013^*^
2	2.646	1.845 – 3.794	< 0.001	1.553	1.040 – 2.321	0.031^*^
≥ 3	5.597	3.765 – 8.321	< 0.001	3.213	2.059 – 5.013	< 0.001^*^
CHA_2_DS_2_-VASc						
0	Reference			Reference		
1	1.319	0.815 – 2.134	0.259	1.218	0.746 – 1.990	0.430
2	2.830	1.767 – 4.532	< 0.001	2.341	1.436 – 3.818	0.001^*^
3	2.473	1.501 – 4.075	< 0.001	1.799	1.061 – 3.050	0.029^*^
≥ 4	5.730	3.713 – 8.842	< 0.001	3.433	2.110 – 5.585	< 0.001^*^
CHA_2_DS_2_-VASc-M						
0	Reference			Reference		
1	1.382	0.786 – 2.431	0.261	1.315	0.740 – 2.334	0.350
2	1.933	1.081 – 3.457	0.026	1.487	0.818 – 2.702	0.193
3	3.927	2.231 – 6.914	< 0.001	2.526	1.398 – 4.562	0.002^*^
≥ 4	5.068	2.971 – 8.643	< 0.001	2.917	1.652 – 5.150	< 0.001^*^
AKI						
CHADS_2_						
0	Reference			Reference		
1	1.971	1.252 – 3.104	0.003	1.654	1.035 – 2.642	0.035^*^
2	2.886	1.811 – 4.600	< 0.001	1.995	1.191 – 3.342	0.009^*^
≥ 3	5.950	3.675 – 9.632	< 0.001	3.991	2.320 – 6.867	< 0.001^*^
CHA_2_DS_2_-VASc						
0	Reference			Reference		
1	1.193	0.667 – 2.132	0.551	1.105	0.613 – 1.990	0.739
2	1.455	0.793 – 2.670	0.225	1.229	0.657 – 2.300	0.517
3	1.693	0.909 – 3.152	0.097	1.335	0.695 – 2.564	0.385
≥ 4	4.094	2.453 – 6.833	< 0.001	2.598	1.449 – 4.657	0.001^*^
CHA_2_DS_2_-VASc-M						
0	Reference			Reference		
1	2.146	0.930 – 4.950	0.073	2.106	0.907 – 4.885	0.083
2	3.026	1.294 – 7.072	0.011	2.515	1.062 – 5.954	0.036
3	3.663	1.564 – 8.579	0.003	2.656	1.104 – 6.390	0.029
≥ 4	6.970	3.143 – 15.458	< 0.001	4.670	2.033 – 10.729	< 0.001
Mechanical ventilation						
CHADS_2_						
0	Reference			Reference		
1	2.063	1.314 – 3.238	0.002	2.098	1.324 – 3.323	0.002^*^
2	2.519	1.564 – 4.056	< 0.001	2.462	1.470 – 4.122	0.001^*^
≥ 3	4.935	3.016 – 8.075	< 0.001	4.767	2.746 – 8.273	< 0.001^*^
CHA_2_DS_2_-VASc						
0	Reference			Reference		
1	0.774	0.427 – 1.403	0.400	0.746	0.410 – 1.359	0.339
2	1.315	0.734 – 2.356	0.356	1.269	0.699 – 2.303	0.433
3	1.836	1.029 – 3.274	0.039	1.763	0.964 – 3.224	0.065
≥ 4	2.912	1.771 – 4.786	< 0.001	2.762	1.570 – 4.859	< 0.001^*^
CHA_2_DS_2_-VASc-M						
0	Reference			Reference		
1	3.697	1.287 – 10.619	0.015	4.005	1.390 – 11.542	0.010^*^
2	5.812	2.013 – 16.777	0.001	6.244	2.150 – 18.130	0.001^*^
3	6.785	2.342 – 19.656	< 0.001	7.070	2.402 – 20.807	< 0.001^*^
≥ 4	10.840	3.889 – 30.213	< 0.001	11.035	3.864 – 31.513	< 0.001^*^

Abbreviations: AKI, acute kidney injury; ARDS, acute respiratory distress syndrome; CI, confidence interval; ES, effect size. ^a^Adjusted for comorbidities including dyslipidemia, atrial fibrillation, chronic kidney disease, chronic respiratory disease, malignancy, tobacco smoking, and opium consumption. *P < 0.01 is significant.

### Secondary endpoints

Table 2 summarizes the results of the three risk scores regarding the defined secondary endpoints. After adjusting for potential confounders in multivariate analysis, these three risk scores were associated with adverse in-hospital clinical features of patients with COVID-19. ARDS occurred in 383 (27.24%) patients, and CHADS_2_ ≥ 1, CHA_2_DS_2_-VASc ≥ 4, and CHA_2_DS_2_-VASc-M ≥ 3 were predictors of ARDS. The cardiac injury was diagnosed in 317 (22.55%) patients, and CHADS_2_ ≥ 1, CHA_2_DS_2_-VASc ≥ 2, and CHA_2_DS_2_-VASc-M ≥ 3 were correlated with cardiac injury. AKI was detected in 177 (12.59%), and CHADS_2_ ≥ 1, CHA_2_DS_2_-VASc ≥ 4, and CHA_2_DS_2_-VASc-M ≥ 2 were linked with the occurrence of AKI. Mechanical ventilation was performed in 168 (11.95%) patients, and CHADS_2_ ≥ 1, CHA_2_DS_2_-VASc ≥ 4, and CHA_2_DS_2_-VASc-M ≥ 1 were associated with the need for mechanical ventilation (Table 2). The prediction performance of these three risk scores concerning the secondary endpoints was almost similar without any statistically significant difference (Figure 3b-e) (Supplementary Table S1). The association between each component of the risk scores with secondary endpoints was depicted in Supplementary Figure S1.

Ultimately, we evaluated the correlation between CHADS_2_, CHA_2_DS_2_-VASc, and CHA_2_DS_2_-VASc-M scores with serum levels of CRP, hs-cTnI, and D-dimer. All three risk scores were significantly correlated with these laboratory parameters; however, the correlation coefficients were all less than 0.5 (ranging from 0.102 to 0.384), which indicates a weak correlation (Supplementary Figure S2).

## Discussion

Drawing from major tertiary hospital data, our results illustrated that the CHADS_2_, CHA_2_DS_2_-VASc, and CHA_2_DS_2_-VASc-M scores could aid prognostication of mortality as well as ARDS, cardiac injury, AKI, and mechanical ventilation in COVID-19 patients, irrespective of the presence of AF. Appraising the weight of each variable included within all risk scores revealed that most of the variables were predictors of unfavorable outcomes in patients with COVID-19. Moreover, we found that all three risk scores were remarkably correlated with CRP, hs-cTnI, and D-dimer serum levels.

Owing to the scarcity of healthcare resources during the COVID-19 pandemic, the need for implementing suitable strategies for equitably allocating the resources has never been timelier.^1^ However, due to the lack of comprehensive data regarding the prognostic impact of different methods, there still exist uncertainties among clinicians to ascertain the prognosis of COVID-19 patients, resulting in increasing the demand for medical resources.^23^ In this perspective, several studies have developed clinical risk estimators to assess the susceptibility for developing unfavorable outcomes.^1,24^ Liang and colleagues^1^ have proposed a clinical risk prediction score consisting of detailed radiological, biochemical, and clinical components for evaluating the prognosis of critically ill patients with COVID-19 at the time of hospital admission. Likewise, Knight et al^24^ created the 4C mortality risk score to stratify the risk of in-hospital mortality in COVID-19 patients. In addition, by employing machine learning techniques, Yadaw et al^25^ have developed a risk score model for predicting COVID-19 mortality. Dissecting the proposed methods by previous studies reveals that most of the included variables are based on radiological data or biomarker levels, which might limit their applicability in clinical practice. On the other hand, the calculation of the CHADS_2_, CHA_2_DS_2_-VASc, and CHA_2_DS_2_-VASc-M scores highly rely on patients’ anamnesis without the need for complex parameters. Hence, it might be much more practical to apply these easily calculable risk scores to timely stratify the risk of mortality in patients with COVID-19.

Dissecting the determinants of mortality in patients with COVID-19, most of the variables of the CHADS_2_, CHA_2_DS_2_-VASc, and CHA_2_DS_2_-VASc-M scores are confirmed to be prognostic risk factors.^4,9-14^ Accordingly, we analyzed the weight of each component for mortality occurrence within the three risk scores, indicating that most of the variables were associated with increased risk of three-month mortality in COVID-19 patients. As the pathophysiological hallmark of the disease, the SARS-COV-2 gains its entry to the targeted cells through the angiotensin-converting enzyme 2 (ACE2) receptor, which is expressed in the kidney epithelium, pancreas, heart, enterocytes, and lungs.^26^ It has been postulated that certain comorbidities are linked with a strong ACE-2 receptor expression, which enhances the viral entry to the host cells. Additionally, a critical casualty of the COVID-19 is propagating the cytokine storm, which may trigger inflammation and unfavorable outcomes in patients with underlying diseases.^4^

As the first component of the three risk scores, CHF has been associated with worse outcomes in COVID-19 patients.^9^ In patients with hypertension, upregulation of the ACE-2 expression has been demonstrated to play a critical role in increasing the fatality of the COVID-19.^4^ In a pooled analysis conducted by Du et al,^10^ patients with hypertension were at 2.17-fold higher risk of mortality. Similarly, elevated levels of ACE-2 receptors and the preexisting defects in the immune system have led to a higher susceptibility for poorer outcomes in patients with DM.^11,27^ In this regard, a recent meta-analysis indicated that patients with DM had significantly higher risks of disease severity and mortality.^11^ In terms of the pre-existence of stroke or TIA, subgroup analysis of a pooled study illustrated that cerebrovascular diseases were associated with higher risks of mortality in COVID-19 patients (relative risk:2.38; *P* < 0.001).^12^ Likewise, the correlation between the presence of vascular disease and increased risk of COVID-19 mortality has been well identified.^13^ In the domain of age affection, Zheng and colleagues^14^ reported that aged over 65 could significantly affect the prognosis of patients with COVID-19. Across gender disparity, the male sex has been enlightened as an essential contributor to COVID-19 progression.^19^ As a result, we also included a modified CHA_2_DS_2_-VASc score to adopt the context of COVID-19 better.

As a critical insight from this study, we revealed that the CHADS_2_, CHA_2_DS_2_-VASc, and CHA_2_DS_2_-VASc-M scores were independent predictors of three-month mortality after adjusting for possible confounders. Our results are in agreement with that of Quisi et al^15^ who found that the CHA_2_DS_2_-VASc score could predict in-hospital mortality in COVID-19 patients. Similarly, Gunduz and colleagues^16^ assessed the potential diagnostic role of CHA_2_DS_2_-VASc, and CHA_2_DS_2_-VASc-M scores in COVID-19 patients, indicating that both risk scores could be applied to stratify the risk of mortality with cut-off values of ≥ 3 scores. In another study, Ruocco et al^17^ elucidated that COVID-19 patients in the highest tertile of CHA_2_DS_2_-VASc scores earned significantly higher risks of mortality (odds ratio:5.65; *P* < 0.001) compared with those in the lowest tertile. Strikingly, the pivotal predictive role of CHA_2_DS_2_-VASc score for mortality in several other diseases has been well identified.^7,8^ In support of this concept, CHADS2 and CHA_2_DS_2_-VASc scores have been employed to predict 1-year all-cause mortality in patients with systolic heart failure.^7^ In addition, Poci et al^8^ demonstrated that the CHADS_2_ score was associated with long-term mortality in patients with acute coronary syndrome (HR:1.38; 95% CI 1.28–1.48).

Interpretation of the ROC analysis revealed that all three risk scores had valuable screening power to determine the prognosis of COVID-19 patients. Besides, the CHA_2_DS_2_-VASc-M score represented even better predictive values compared to the other ones, although it may lack clinical relevance due to the small statistical differences. Consistent with this notion, Caro-Codo’n et al^23^ reported AUC of 0.788, 0.794, and 0.820 for CHADS_2_, CHA_2_DS_2_-VASc, and CHA_2_DS_2_-VASc-M scores to predict mortality among COVID-19 patients. Likewise, another investigation showed that the CHA_2_DS_2_-VASc score had a valuable prognostic ability for predicting ICU admission and mechanical ventilation in low-risk COVID-19 patients.^28^ Furthermore, in a study that evaluated plausible predictors of in-hospital mortality on 694 COVID-19 patients, both CHA_2_DS_2_-VASc-M over CHA_2_DS_2_-VASc scores had valuable discriminative abilities, with higher AUC values for CHA_2_DS_2_-VASc-M.^29^ Taken together, interpretation of our findings in accompany with previous studies reveal that all three risk scores could serve as a simplified means of rapid assessment, which could result in effectively guiding high-risk patients with COVID-19.

As a distinctive feature of this investigation, our analysis reinforced the CHADS_2_, CHA_2_DS_2_-VASc, and CHA_2_DS_2_-VASc-M scores as potential tools to predict cardiac injury in COVID-19 patients. The ROC analysis confirmed the prognostic ability of these risk scores for cardiac injury. As an endorsement for this analysis, we also indicated that all three risk scores were significantly correlated with serum levels of hs-cTnI. These findings agree with a previous study on patients with acute myocardial infarction, demonstrating that the incidence of cardiac events was higher as the CHA_2_DS_2_-VASc score increased.^6^ Regarding the plausible pathophysiological hallmarks of cardiac injury in COVID-19 patients, the unmasking of underlying cardiovascular disease, susceptibility for developing acute coronary syndrome and myocarditis, and propagation of the cytokine cascade has been blamed.^30,31^ Interestingly, most of the components of the three risk scores are recognized to be associated with increased risks of developing cardiac injury in COVID-19 patients.^32-36^ The essential impact of the cardiac injury on developing poor outcomes has been addressed by previous efforts.^32-34^ Besides, as the crucial representer of cardiac injury, elevation of troponin levels has been associated with increased risk of COVID-19 progression, although this elevation is reported to be delayed, particularly one week preceding the death.^37-39^ Therefore, it seems critical to apply the CHADS_2_, CHA_2_DS_2_-VASc, and CHA_2_DS_2_-VASc-M scores to identify high-risk patients at earlier stages.

Lastly, we indicated that all three risk scores could predict ARDS, mechanical ventilation, and AKI in patients with COVID-19. In addition, these risk scores had remarkable associations with CRP and D-Dimer. Notably, several variables within the three risk scores have been demonstrated to predict ARDS, AKI, and mechanical ventilation utilization in COVID-19 patients.^10,11,40-43^ Investigating the essential pathological pathways reveals that virus-induced cytopathic effects on the podocytes cells in the kidney and downregulating the expression of ACE-2 in lung cells could promote kidney and lung injury, respectively.^26,44,45^ The SARS-COV-2 mediates its effect on the lungs and kidneys through the initiation of the hyperinflammatory state and diffuse intravascular coagulopathy, which is associated with elevated levels of D-dimer, CRP, and cardiac enzymes.^26,44,46-48^ These observations could raise the possibility that the CHADS_2_, CHA_2_DS_2_-VASc, and CHA_2_DS_2_-VASc-M scores may reflect a remarkable pro-inflammatory and hypercoagulability state in patients with COVID-19, resulting in developing ARDS, AKI, and eventually mortality in COVID-19 patients.

All in all, our findings illustrated that rather than individual variables of the CHADS_2_, CHA_2_DS_2_-VASc, and CHA_2_DS_2_-VASc-M scores, the total scores revealed valuable prognostic performance for unfavorable outcomes in COVID-19 patients. Even though this is an observational study with its inherent biases, it supports the statement that all three risk scores could be integrated into the overall therapeutic strategy to guide the COVID-19 management more accurately. From the perspective of clinicians, these results are of utmost importance, given that employing an easily calculable tool for stratifying the prognosis of COVID-19 patients could aid in implementing suitable strategies for patients at higher risks of disease progression.

The current investigation has addressed the predictive role of the CHADS_2_, CHA_2_DS_2_-VASc, and CHA_2_DS_2_-VASc-M scores through a three-month follow-up. Our analysis provides new insights into the existing literature by evaluating whether these scores could be used to estimate the susceptibility to ARDS, cardiac injury, AKI, and mechanical ventilation in patients with COVID-19. Moreover, the sample size of the study is notably larger than most of the previous studies. On the contrary, we would like to emphasize the limitations of the study. First, this is a single-center observational study, which has its inherent limitations; thus, further longitudinal multicenter studies should be conducted to confirm these results more accurately. Also, we could not ascertain the thromboembolic event as a secondary endpoint. However, with respect to the findings of a recent study, the discussed risk scores did not predict thromboembolic events in patients with COVID-19, emphasizing that all three risk scores could predict poor outcomes in COVID-19 patients, regardless of the development of thromboembolic events.^23^

## Conclusion

In summary, we have endorsed an early pragmatic method to stratify the risk of poor outcomes in COVID-19 patients. Our analysis demonstrated that the CHADS_2_, CHA_2_DS_2_-VASc, and CHA_2_DS_2_-VASc-M scores could predict mortality, ARDS, cardiac injury, AKI, and mechanical ventilation in COVID-19 patients, irrespective of the presence of AF. Also, we found that all three risk scores were remarkably correlated with serum levels of CRP, hs-cTnI, and D-dimer. Our strategy regarding the employment of these three risk scores has advantages over previously designated risk scores in that it relies on patients’ anamnesis, can be performed at admission, and is not dependent on complex laboratory or radiological parameters. Given that fostering the optimal approach to mitigate the imposed burden of COVID-19 necessities suitable prognostication of high-risk patients, our findings could have a pivotal clinical impact on the management of COVID-19 patients.

## Acknowledgments

We acknowledge all healthcare workers involved in the diagnosis and treatment of patients in Sina Hospital. We are indebted to the Research Development Center of Sina Hospital for its support.

## Funding

This work was supported by the [Tehran University of Medical Sciences] under Grant [99-1-101-47211]. No funding was received to assist with the preparation of this manuscript.

## Ethics approval

The research complied with the principles of the 1975 declaration of Helsinki. The protocol of this study was approved by the Ethics Committee of Tehran University of Medical Sciences (IR.TUMS.VCR.REC.1399.005). All participants or their legal guardians gave written informed consent before inclusion in the study. The study protocol has been priorly approved by the Institution’s ethics committee on research on humans.

## Competing interests

The authors report no conflicts of interest.

## Supplementary file


Supplementry file contains Table S1, Figure S1 and Figure S2.
