# Supplementary material for: Prognostic significance of CHADS2 and CHA2DS2-VASc scores to predict unfavorable outcomes in hospitalized patients with COVID-19
Source: J Cardiovasc Thorac Res. 2022 Mar 14;14(1):23–33. doi: 10.34172/jcvtr.2022.07 (PMC9106940; doi:10.34172/jcvtr.2022.07)
Supplement: Supplementary file 1 — Supplementry file contains Table S1, Figure S1 and Figure S2. [file jcvtr-14-23-s001.pdf]

Supplementary Material

Table S1. Prediction performance $CHADS_2$, CHA_2DS_2-VASc, and CHA_2DS_2-VASc-M for the study endpoints

Endpoint	Risk score	AUC	95% CI	P-value comparing risk scores
Three-month mortality	$CHADS_2$	0.668	0.635 - 0.701	0.250
	CHA_2DS_2-VASc	0.668	0.634 - 0.702	
	CHA_2DS_2-VASc-M	0.681	0.648 - 0.714	
ARDS	$CHADS_2$	0.579	0.547 - 0.611	0.932
	CHA_2DS_2-VASc	0.576	0.543 - 0.610	
	CHA_2DS_2-VASc-M	0.579	0.546 - 0.612	
Cardiac injury	$CHADS_2$	0.650	0.616 - 0.683	0.107
	CHA_2DS_2-VASc	0.671	0.638 - 0.705	
	CHA_2DS_2-VASc-M	0.667	0.634 - 0.701	
AKI	$CHADS_2$	0.662	0.620 - 0.704	0.369
	CHA_2DS_2-VASc	0.649	0.604 - 0.694	
	CHA_2DS_2-VASc-M	0.664	0.622 - 0.706	
Mechanical ventilation	$CHADS_2$	0.642	0.599 - 0.685	0.120
	CHA_2DS_2-VASc	0.634	0.588 - 0.681	
	CHA_2DS_2-VASc-M	0.659	0.617 - 0.700	

Abbreviations: AKI, acute kidney injury; ARDS, acute respiratory distress syndrome; AUC, area under the curve; CI, confidence interval.

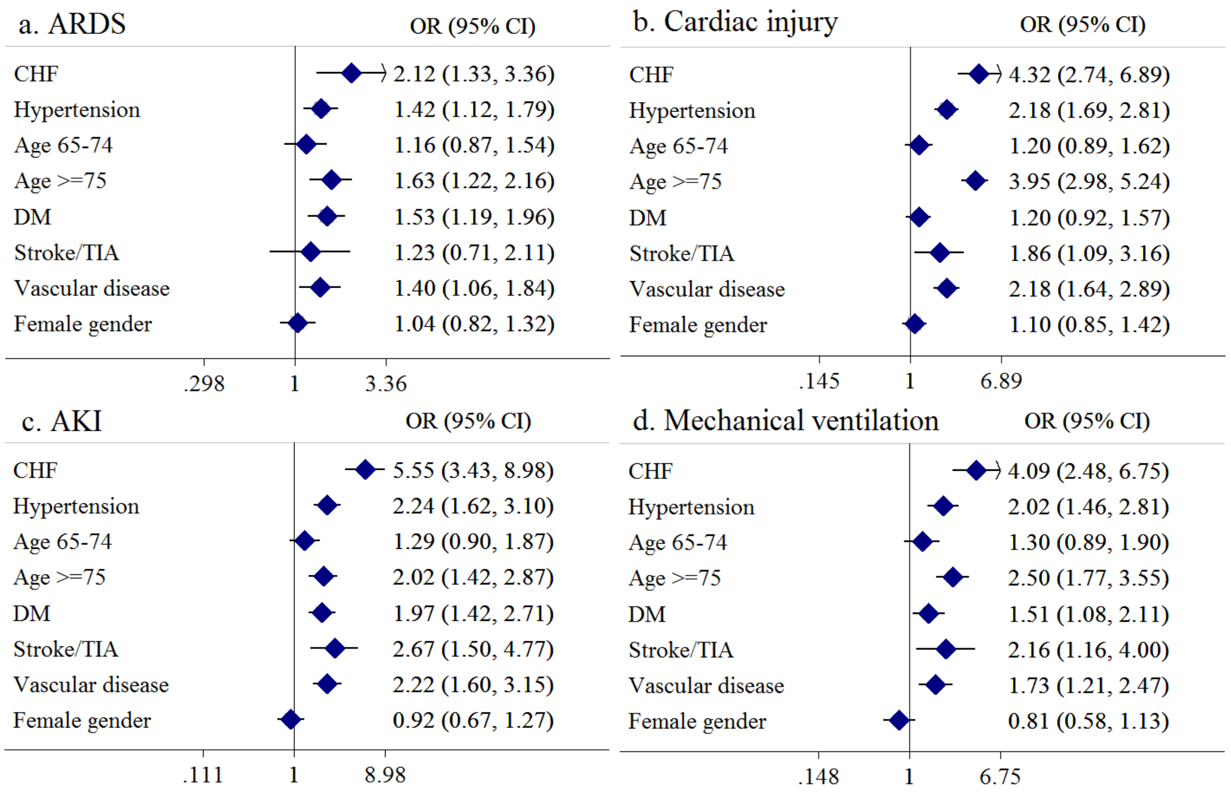

Figure S1. Forrest plot of odds ratios for individual CHA_2DS_2-VASc components regarding (a) ARDS, (b) Cardiac injury, (c) AKI, and (d) Mechanical ventilation. Abbreviations: AKI, acute kidney injury; ARDS, acute respiratory distress syndrome.

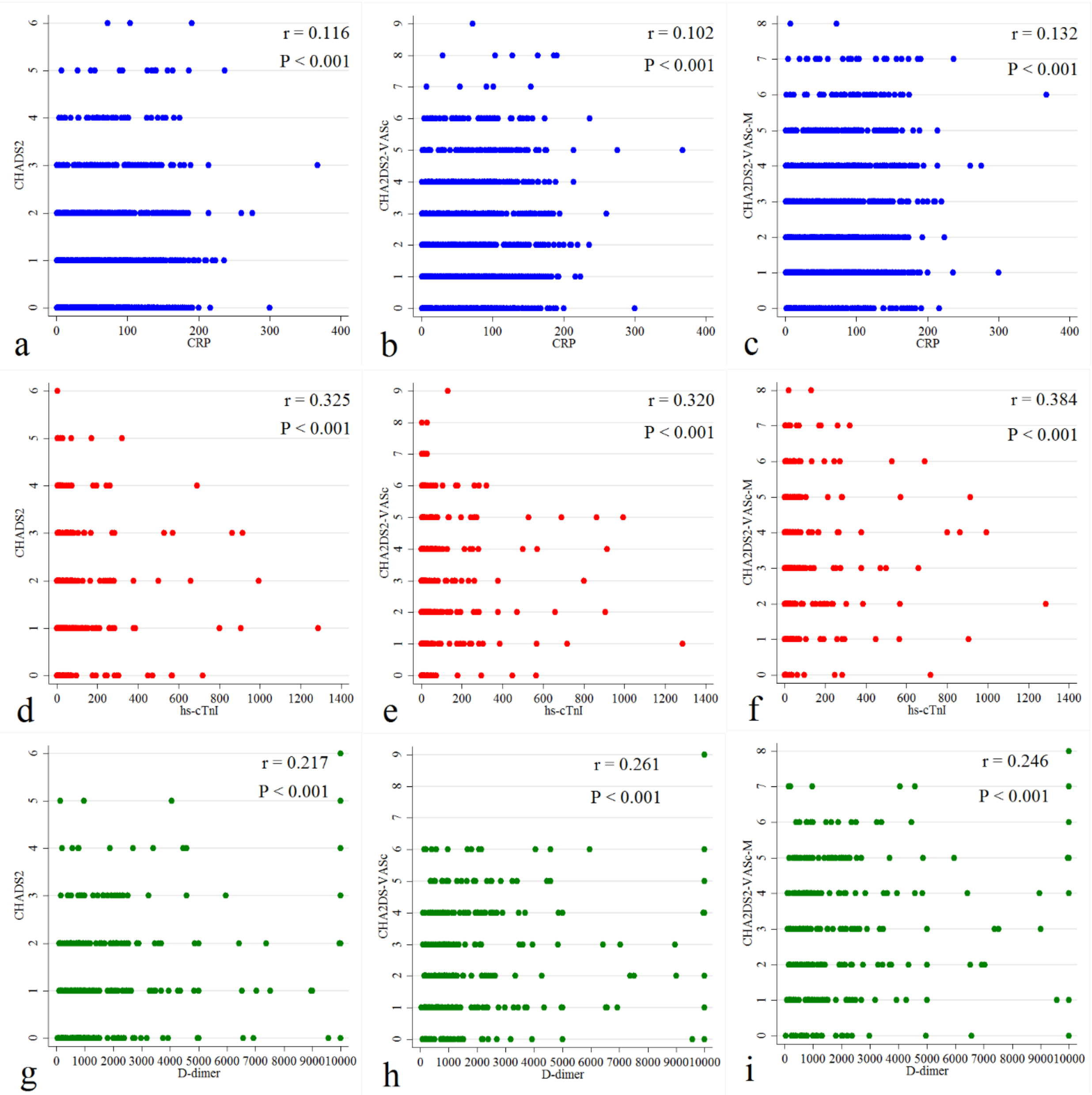

Figure S2. Correlation between $CHADS_2$, CHA_2DS_2-VASc and CHA_2DS_2-VASc-M scores and CRP (mg/L), hs-cTnI (pg/mL), and D-dimer (mg/L). Abbreviations: CRP, C-reactive protein; hs-cTnI, highly sensitive cardiac troponin I.
